# Neutralization of X4- and R5-tropic HIV-1 NL4-3 variants by HOCl-modified serum albumins

**DOI:** 10.1186/1756-0500-3-155

**Published:** 2010-06-02

**Authors:** Svenja Polzer, Melanie van Yperen, Martin Kirst, Birco Schwalbe, Heiner Schaal, Michael Schreiber

**Affiliations:** 1 Bernhard-Nocht Institute for Tropical Medicine, Department of Virology, Hamburg, Germany; 2 Institute for Virology, Heinrich-Heine-University of Duesseldorf, Germany; 3 Current address: Cephalon GmbH, Martinsried, Germany

## Abstract

**Background:**

Myeloperoxidase (MPO), an important element of the microbicidal activity of neutrophils, generates hypochlorous acid (HOCl) from H_2_O_2 _and chloride, which is released into body fluids. Besides its direct microbicidal activity, HOCl can react with amino acid residues and HOCl-modified proteins can be detected *in vivo*.

**Findings:**

This report is based on binding studies of HOCl-modified serum albumins to HIV-1 gp120 and three different neutralization assays using infectious virus. The binding studies were carried out by surface plasmon resonance spectroscopy and by standard ELISA techniques. Virus neutralization assays were carried out using HIV-1 NL4-3 virus and recombinant strains with CXCR4 and CCR5 coreceptor usage. Viral infection was monitored by a standard p24 or X-gal staining assay. Our data demonstrate that HOCl-modified mouse-, bovine- and human serum albumins all bind to the HIV-1 NL4-3 gp120 (LAV) glycoprotein in contrast to non-modified albumin. Binding of HOCl-modified albumin to gp120 correlated to the blockade of CD4 as well as that of V3 loop specific monoclonal antibody binding. In neutralization experiments, HOCl-modified serum albumins inhibited replication and syncytium formation of the X4- and R5-tropic NL4-3 isolates in a dose dependent manner.

**Conclusions:**

Our data indicate that HOCl-modified serum albumin veils the binding site for CD4 and the V3 loop on gp120. Such masking of the viral gp120/gp41 envelope complex might be a simple but promising strategy to inactivate HIV-1 and therefore prevent infection when HOCl-modified serum albumin is applied, for example, as a topical microbicide.

## Background

An important event in HIV-1 infection is the step-by-step binding of the external envelope complex, the gp120/gp41 trimer, to (i) CD4 [1] and to (ii) a family of seven-transmembrane chemokine receptors including CXCR4 and CCR5 which are the two major coreceptors [2] on the cellular membrane as well as to (iii) heparan sulfate structures [3]. HIV-1 entry can therefore be inhibited by heparan sulfate [4], its analogs [5] or other synthetic polyanions [6], ligands for CD4 [7], CXCR4 [8] or CCR5 [9], and by factors that bind to gp120 like neutralizing antibodies [10]. Since HIV-1 variability is prodigious, viral escape to all these antiviral substances has been documented and therefore there is a pressing need to find new strategies to efficiently block HIV-1 infection.

In 1992, Klebanoff and coworkers [11] showed that stimulated polymorphonuclear (PMN) cells released an unknown factor which neutralized HIV-1. PMN from patients with hereditary myeloperoxidase (MPO) deficiency indicated that the antiviral activity was correlated with the presence and the release of the enzyme into cell culture medium. Adding MPO to PMN-(MPO-deficient) cell cultures restored the ability to block HIV-1 infection [11,12]. In addition to MPO, the presence of two other factors, H_2_O_2 _and chloride, was absolutely necessary to observe the antiviral activity in cell culture supernatants of stimulated PMN cells. Since the enzyme MPO catalyzes the reaction between H_2_O_2 _and chloride to generate HOCl (bleach), Klebanoff and coworkers [12] suggested that this product of the MPO/H_2_O_2_/halide system was directly responsible for HIV inactivation. A clue as to the substrate of the MPO/H_2_O_2_/halide system was provided by the detection of HOCl-modified proteins in human tissue by a specific monoclonal antibody (clone 2D10G9) [13]. This antibody recognized an HOCl-modified protein in glomerular and tubulointerstitial inflammatory and fibrotic lesions and its binding was inhibited by HOCl-modified human serum albumin (mHSA) [14,15].

Recently we demonstrated that mHSA was active against West Nile virus (WNV) [16]. Low doses of HOCl-modified human serum albumin (mHSA) inactivated WNV entry into VeroB4 cells *in vitro *and we observed an interaction between mHSA and the domain III of the WNV external envelope. Similar to WNV, HIV-1 also enters its target cell following membrane fusion of the viral envelope and the plasma membrane and it is conceivable that enveloped viruses share common functions to promote membrane-membrane contact to allow binding of cell membrane-anchored receptors.

Here we report, that mHSA, mBSA (bovine) and mMSA (mouse) bind to recombinant HIV-1 gp120 of the laboratory strain NL4-3. Binding of HOCl-modified serum albumins to gp120 also inhibited binding of recombinant CD4 (rCD4) and V3 loop-specific antibodies. All three HOCl-modified serum albumins inhibited viral infection and syncytium formation in a dose dependent manner.

## Method

### Viruses, Cell Lines, Env expression plasmids, and HIV Reagents

HIV-1 strain NL4-3, an X4-monotropic laboratory isolate, was used for virus inhibition tests. HeLa-P4 cells expressing human CD4, CXCR4 were kindly provided by Matthias T. Dittmar, University Heidelberg. GHOST-CXCR4 cells, antibody sera, monoclonal antibody, and gp41 (ARP671, spanning the entire extracellular domain of HIV-1 gp41_MN_) were obtained from the European Vaccine Against AIDS Programme (EVA, Potters Bar, UK). Recombinant HIV-1 gp120 (LAV, CXCR4 monotropic, identical to NL4-3 gp120, glycosylated) and human rCD4 (soluble form, transmembrane region removed, glycosylated) was obtained from Protein Sciences Corporation, Meriden, CT, USA. These commercially proteins were expressed in insect cells by a baculovirus vector system, secreted in the serum-free cell culture medium and purified under non-denaturing conditions. The *env *expression plasmid pSVATGrev carries a subgenomic HIV NL4-3 fragment coding for *rev*, *vpu *and *env *under the transcriptional control of the SV40 early promotor. The plasmid sequence can be obtained upon request. Construction of NL4-3 mutants with R5X4 and R5 tropism and mutants lacking N-glycosylation site g15 was carried out as described earlier [17].

### Modification of serum albumins by HOCl

The HOCl reaction was carried out as described in the literature [18] with modifications. In brief, freshly prepared HOCl was added to an aqueous solution of serum albumin (1 gr/liter phosphate buffered saline, pH 7.5). The concentration of HOCl was monitored by UV spectroscopy (ε = 375 mol/cm liter). Serum albumin was treated with HOCl at different molar ratios (serum albumin:HOCl, 1:10, 1:20, 1:100, 1:200, 1:500, 1:1000, 1:10000). After incubation for 30 min at RT, remaining HOCl was inactivated by adding the antioxidant 2 aminoethanesulfonic acid (taurine, final concentration 5 gr/l). The mixture was incubated again for 30 min at RT. Purification of the HOCl-modified serum albumin and the elimination of remaining 2 aminoethanesulfonic acid was achieved by concentrating the reaction mixture to a final volume of 50 ml by ultrafiltration (GE Healthcare, hollow fibre column UFP-10-C 3X2MA, membrane nominal-molecular-weight cutoff 10 kD) and then further purified by a continuous ultrafiltration process using the QuixStand^® ^apparatus (GE Healthcare). A total volume of 20 litres of water was slowly added over a time period of 48 h to purify the mHSA product which was continuously pumped over the hollow fibre column at a maximum pressure of 15 PSI. After this ultrafiltration process the solution (400 ml) was again concentrated to a final volume of 50 ml. The final yield of HOCl-modified serum albumin was between 250-500 mg/50 ml (i.e. 25-50%).

### ELISA binding studies

ELISA plates (Nunc, MaxiSorb) were coated with gp120 (3 μg/ml in carbonate buffer 0.1 M NaHCO_3_, 0.1 M Na_2_CO_3_, pH 9.6). To each well of a 96-well plate 100 μl of the gp120 solution was added and the plates were sealed with adhesive foil (Sarstedt) and incubated at 8°C overnight. Subsequently 250 μl of a BSA solution (20 μg/ml PBS) was added to each well and the plates incubated for 1 h at room temperature. After blocking, the plates were washed 5-times with PBS containing 0.05% Tween 20 (PBST). Modified serum albumin was added to the wells in a total volume of 50 μl PBS. After incubation for 30 min. rCD4 or antibody was added to each well in a total volume of 50 μl PBS. The plates were incubated for 1 h at room temperature and then washed 5-times with PBST. To detect gp120-bound rCD4 we used the anti-CD4 mouse monoclonal antibody NCL-CD4-1F6 (Novocastra). Bound anti-CD4 antibody was detected by a secondary anti-mouse HRP-antibody conjugate diluted 1:500 in PBS (BioRad). Bound gp120-specific monoclonal antibody was monitored directly using the anti-mouse-HRP-antibody conjugate (BioRad). After binding of the secondary antibody, the plates were washed 5 times with PBST and 5 times with PBS. To each well 100 μl of a commercially available one-component ready to use solution of 3,3',5,5'-tetramethylbenzidine (TMB, Sigma Aldrich) was added, the reaction stopped with 1 M H_2_SO_4_, and the plate read at 450 nm.

### Biosensor binding studies

Purified recombinant LAV gp120 (1 μg/100 μl 10 mM NaOAc pH 4.0) was immobilized by NHS/EDC coupling on a dextran coated, CH-activated C5 sensorchip (Biacore, Sweden) at densities yielding 3500-5000 response units (RU). Surface plasmon resonance was carried out using a Biacore 1000. Binding to immobilized proteins diluted in HBS-EP buffer (Biacore, Sweden) was tested at various concentrations at a flow rate of 5 μl/min for a period of 6 min.

### Cell proliferation assays

To each well of a 96-well plate, containing 10.000 CD4^+ ^HeLa-P4 cells inhibitor and control proteins were added at various concentrations and the cells were incubated for 48 h. Thereafter the medium was changed and 10 μl MTT (3-(4, 5-dimethylthiazolyl-2)-2, 5-diphenyltetrazolium bromide) solution (5 mg/ml MTT dissolved in PBS pH 7.4) was added. The plates were incubated at 37°C for 4 h after which the medium was removed and 100 μl of lysis buffer (9.94 ml DMSO, 60 μl HCl, 1 g SDS) added to each well. The plates were incubated for 10 min at 37°C. Absorbance was measured at 560 nm with background subtraction at 630 nm. Cells were cultured in Dulbecco's Modified Eagle Medium (DMEM, Gibco) including 10% fetal calf serum (FCS, Biochrom KG, Berlin, Germany).

DMEM is composed of D-glucose, phenol red, inorganic salts, vitamins and a mixture of 15 free amino acids containing the HOCl-sensitive amino acids Arg (0.4 M), Cys (0.2 M), His (0.2 M), Lys (0.8 M), Met (0.2 M), Phe (0.4 M) and Tyr (0.4 M).

### Virus inhibition

(a) GHOST-CXCR4 cells were infected with 1000 TCID (Tissue Culture Infective Dose) of the X4-tropic laboratory strain NL4-3. To study inhibition, virus was given to the cells after preincubation of cells with modified serum albumin for 30 min. Cell culture supernatants were tested on day 5 for p24-antigen by a standard p24-antigen ELISA. GHOST cells were cultured in DMEM (Gibco) including 10% FCS (Biochrom KG). (b) TZM-bl cells were infected with virus supernatant representing an infectious dose of 500 foci/96-well. Viral entry into TZM bl cells causes the expression of β-galactosidase, which can be monitored 2 days after infection. To detect β-galactosidase-expressing cells, the cell culture supernatant was removed and the cell layer was treated with 0.25% glutaraldehyde in phosphate-buffered saline (PBS, 5 min, RT) and washed three times with PBS. To stain infected cells, 100 μl of the X-gal solution (0.5 mg X-gal [5-bromo-4-chloro-3-indolyl-β-D-galactopyranoside]/ml, 1 mM MgCl_2_, 3 mM potassium ferrocyanide, 3 mM potassium ferricyanide in PBS) was added to each well and the plates were incubated at 37°C. The staining reaction was monitored by microscopy. Finally, the cell layer was washed again with PBS and blue foci were counted.

### Inhibition of syncytium formation

GHOST CXCR4 cells (10^4 ^cells per 96-well) cultured in DMEM/10% FCS were infected with 1000 TCID of the HIV laboratory isolates NL4-3 and were also infected in the presence of 50 μg mHSA per ml cell culture medium (DMEM/10% FCS). Virus and mHSA were added to the cells simultaneously. The cultures were assessed microscopically 5 days after infection by visualization of induced syncytia and cell layer destruction. To visualize syncytia, cell layers were treated with 3% formalin for 10 min to inactivate virus and then fixed with methanol (Solution 1 of the Azur B and Eosin G based Hemacolor^® ^rapid staining set, Merck, Germany) for 5 min. After fixation, cells were stained with color reagent red (solution 2 of the Hemacolor^® ^staining set) for 30 sec followed by a staining with color reagent blue (solution 3 of the Hemacolor^® ^staining set) for 30 sec. Alternatively, HeLa-P4 cells, cultured in DMEM/10% FCS, expressing human CD4, CXCR4 were transfected with 1 μg pSVATGrev plasmid. This plasmid expresses the gp160 protein precursor, which is processed and expressed on the outside of the cellular membrane as the functional gp120/gp41 complex. Cells were transfected for induction of syncytia and mHSA was given to the cells 30 min after transfection. Syncytium formation was assessed after 28 h by standard phase contrast microscopy.

## Results

### Generation of HOCl-modified serum albumins

**Figure 1:**
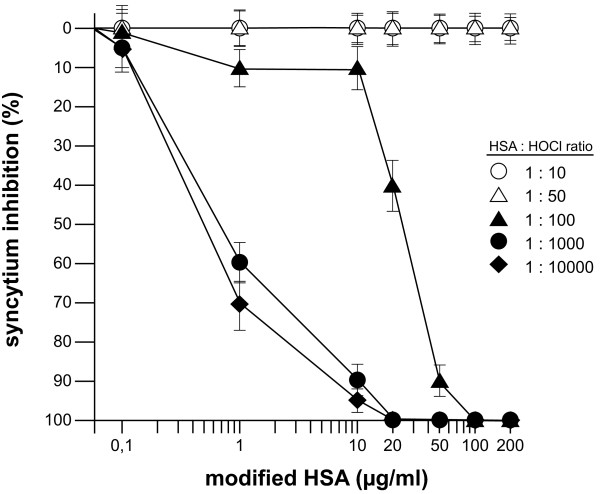
**Effect of HOCl on HSA and the ability to inhibit syncytium formation**. To test the transformation effect of HOCl on HSA, mHSA samples were produced, which were generated using different HOCl:HSA ratios. The different mHSA preparations were tested for the inhibition of gp120/gp41-induced syncytia. HeLa-P4 cells were transfected with pSVATGrev plasmid expressing the NL4-3 (LAV) *env*. Syncytia were visualized by a standard staining procedure (Hemacolor^®^, Merck) and the nuclei of fused cells were counted. Shown are the averages of 6 wells.

In a first experiment we tested different samples of the HOCl-modified HSA, which were generated by using different HOCl concentrations, in a syncytium inhibition assay. In HIV-1 cell cultures, infected cells fuse with other, non-infected CD4^+ ^target cells. Cell-cell fusion is known as syncytium formation. Syncytium formation is the result of the binding of gp120, which is present on membranes of the infected cells, to the receptors on the target cells and subsequent insertion of the gp41 N-terminus into the host membrane. This gp120-receptor binding event promotes recurring cell-cell fusion which leads to the generation of large syncytia. The mHSA samples generated with low HOCl concentrations (molar ratios HSA:HOCl 1:10 and 1:50) showed no inhibitory effect on syncytium formation (Figure 1). At higher concentrations (molar ratio 1:100) an inhibitory effect of mHSA was detected with 90% inhibition at a concentration of approximately 50 μg/ml. The mHSA samples produced at molar ratios 1:1000 and 1:10.000 showed strong inhibition with 90% at 10 μg/ml and complete inhibition of gp120-induced syncytium induction at 20 μg/ml. Thus, HSA treated with higher concentrations of HOCl is transformed into an antiviral form in contrast to HSA samples treated with low HOCl doses.

### Binding of gp120 to HOCl-modified serum albumins

**Figure 2:**
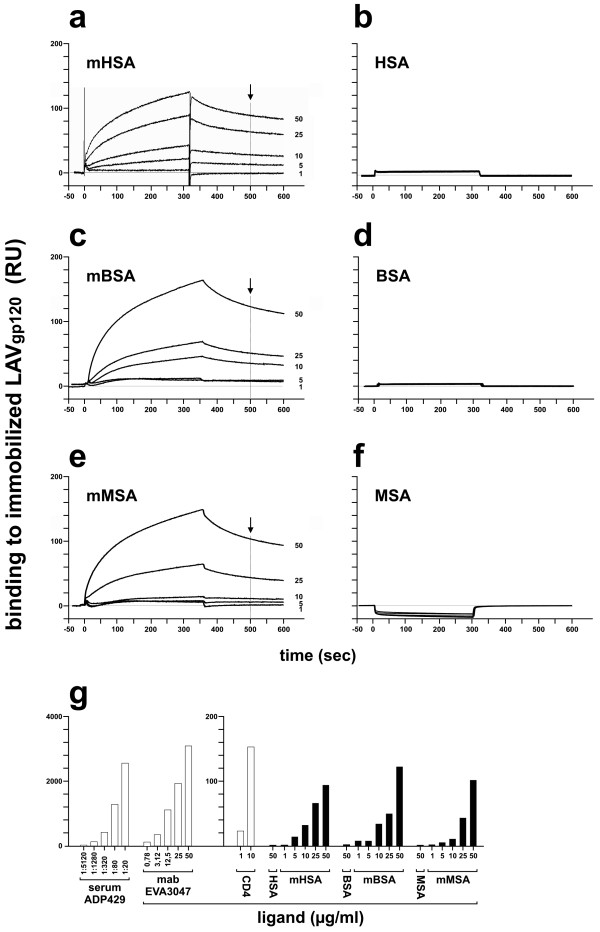
**Binding of HOCl-modified serum albumins to gp120**. (a-f) Binding of HSA, BSA, MSA and HOCl-modified albumins mHSA, mBSA, mMSA (protein:HOCl molar ratio 1:1000) to LAV-gp120 was tested by surface plasmon resonance spectroscopy (Biacore 1000, GE Healthcare). Purified LAV gp120 (10 μg/ml, Protein Sciences Corporation) was covalently linked using EDC/NHC to a dextran coated, CH-activated sensor chip (CM5, GE Healthcare). After blocking with ethanolamine the biosensor was washed three times with 30 μl 100 mM NaOH and 30 μl 100 mM HCl (flow rate 5 μl/min). Proteins were tested for binding at 1, 5, 10, 25 and 50 μg/ml at a flow rate of 5 μl/min. (g). Binding of mHSA, mBSA and mMSA (values marked by the black arrow in Figure 1a, 1c and 1e) was compared to the binding of CD4, and gp120-specific antibodies to a LAV gp120 coated CM5 biosensor. ADP429: anti-serum, derived from HIV-1 SF2 gp120-immunized sheep (AIDS reagent project, NIBSC, UK). EVA3047: monoclonal antibody, derived from IRIQRGPGRAFTIGC-peptide immunized mice (AIDS reagent project, NIBSC, UK).

Since a 1000-fold molar HOCl excess was sufficient to transform HSA into a viral entry inhibitor we generated HOCl-modified samples of three serum albumins from human, mouse and bovine by using a 1000-fold excess of HOCl. Using these three serum albumin preparations, we studied binding of mHSA, mBSA and mMSA to LAV (NL4-3) gp120 by surface plasmon resonance spectroscopy (SPR) (Figure 2a, c, e). The experiment demonstrated that serum albumin modified by a 1000-fold molar excess of HOCl bind to the immobilized gp120. No binding of mHSA, mBSA and mMSA to gp41 immobilized on a biosensor (binding < 5 RU, data not shown) was observed. Thus, binding was specific for gp120 envelope. Bound protein was removed from the gp120-biosensor by treatment with 100 mM HCl and 100 mM NaOH. This regeneration procedure retained the ability of immobilized gp120 to bind rCD4 and V2- and V3-loop specific antibodies. Also no binding to gp120 was measured using normal, non-modified HSA, BSA and MSA (Figure 2b, d, f). In these control experiments response units (RU) in SPR were also below 5 RU. Binding of HOCl-modified proteins was compared to gp120-binding of rCD4 and gp120-specific antibodies (Figure 2g). At 50 μg/ml the modified proteins showed between 50-70% of the gp120 binding activity observed with rCD4 at 10 μg/ml. Binding of antibodies generated by immunization with CHO-derived SF2-gp120 (Serum ADP429) or by a V3 loop peptide (IRIQRGPGRAFTIGC) (mab EVA 3047) showed binding responses at 1:80 dilution (Serum ADP429) or 25 μg/ml (mab EVA 3047) about 30 40 times as high as the HOCl-modifed serum albumins at 50 μg/ml.

### Blocking of rCD4 and antibody binding to gp120

**Figure 3:**
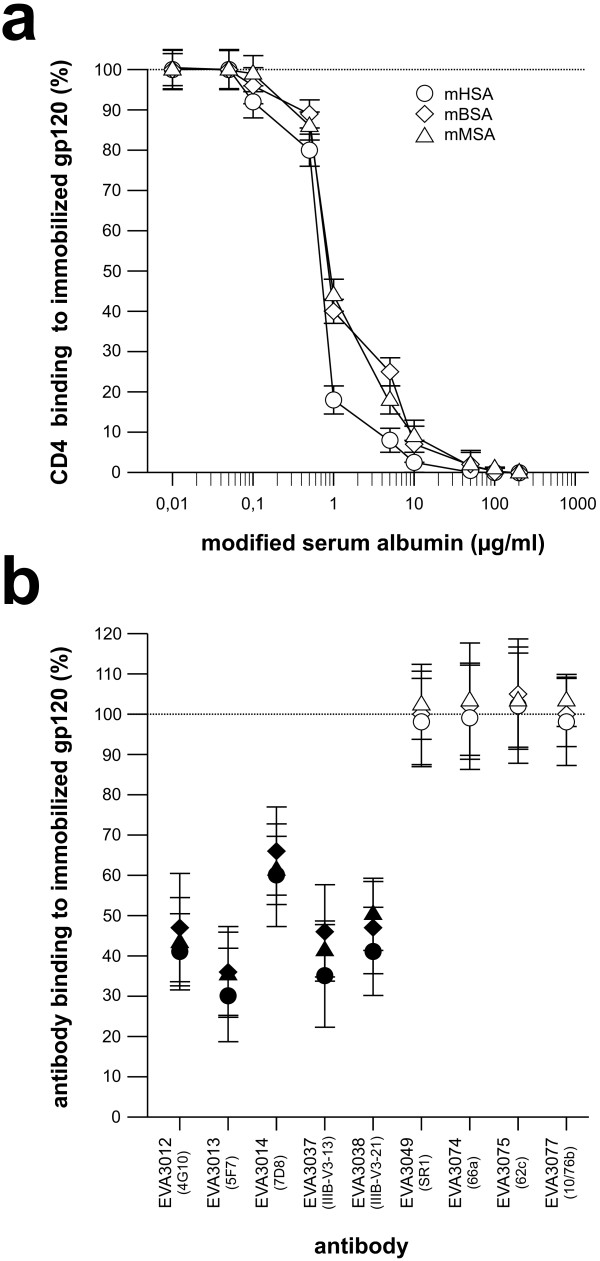
**Inhibition of CD4 and antibody binding to gp120**. Binding of CD4 or antibody, in the presence of mHSA, mBSA and mMSA, to LAV gp120 was tested by ELISA. ELISA plates were coated with LAV gp120 (1 μg/ml, Protein Sciences Corporation) over night. After gp120 binding, the wells were coated using BSA (20 mg/ml). Modified serum albumin was added to the wells and (a) CD4 (1 μg/ml, Protein Sciences Corporation) or (b) antibody diluted in phosphate buffered saline (PBS) pH 7.5, was added 30 min later. After 1 hour, the plates were washed 5-times with PBS. Binding of CD4 to gp120 was carried out at various mSA concentrations. Gp120-bound CD4 was detected by a CD4 specific mouse monoclonal antibody (NCL-CD4-1F6, Novocastra) followed by a staining with an anti-mouse HRP-antibody conjugate (BioRad). Shown are the averages of triplicate wells. Binding of V2- and V3-loop-specific monoclonal antibodies (mab) was carried out in the presence of 10 μg/ml of mHSA. Gp120-bound antibody was detected using an anti-mouse-HRP-antibody conjugate (BioRad). Antibodies were designated according to the repository reference of the Centralized Facility for AIDS Reagents. In brackets: originators antibody designation. All measurements were carried out in triplicates. White symbols: V2 loop specific mab. Black symbols: V3 loop-specific mab.

Next we investigated whether binding of rCD4 or V2- and V3-loop specific antibodies to gp120 was influenced by HOCl-modified serum albumin (Figure 3). These binding studies showed that mHSA, mBSA and mMSA completely inhibited binding of rCD4 to LAV gp120 at concentrations above 10 μg/ml (Figure 3a). In control experiments, neither binding of the anti-CD4-1F6 monoclonal antibody to CD4 nor binding of the secondary HRP-conjugated antibody was inhibited by HOCl-modified serum albumins (data not shown). In addition to rCD4, binding of V3 loop-specific monoclonal antibodies was reduced but not completely inhibited at 10 μg/ml (Figure 3b). In contrast to binding of V3 loop antibody, binding of V2 loop antibody was not inhibited by mHSA, mBSA or mMSA.

### Virus neutralization by HOCl-modified serum albumins

**Figure 4:**
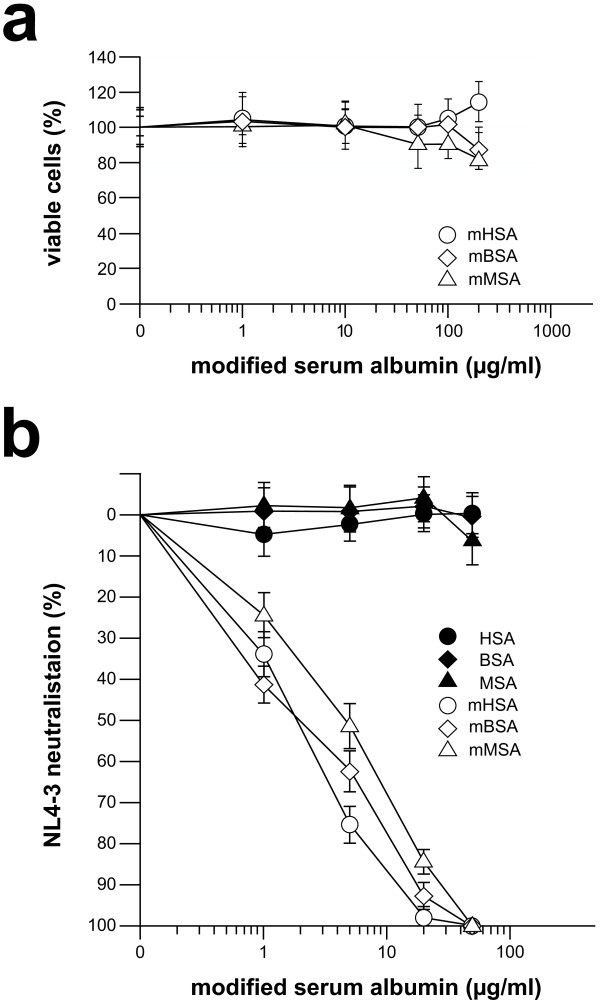
**Inhibition of NL4-3 infection**. (a) Effects of HOCl-modified serum albumins (molar ratio 1:1000) on cell growth. HeLa-P4 cells were cultured in the presence of MTT and various concentrations of mHSA, mBSA and mMSA (white symbols, 1, 5, 20 and 50 μg per ml; Black symbols, non-modified serum albumins). Cells were seeded at 10^4 ^cells per well in 96-well plates in triplicate. After adding the yellow tetrazolium salt MTT, metabolically active cells produced purple formazan. The amount of formazan was measured at 530 nm and is a correlate for cell viability and proliferation. (b) NL4-3 neutralization by HOCl-modified serum albumins (molar ratio 1:1000). HeLa-P4 cells were infected with 1000 TCID of the HIV-1 laboratory strain NL4-3. Shown are the averages of triplicate wells. Infection was monitored 5 days after infection by a standard p24 antigen ELISA.

**Figure 5:**
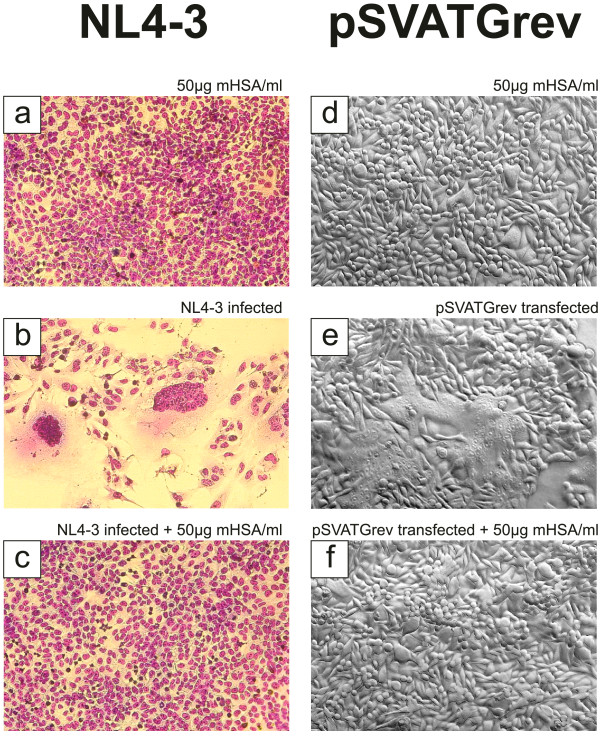
**Inhibition of NL4-3 and gp120-induced syncytium formation**. (a-c) Inhibition of virus induced syncytium formation. GHOST-CXCR4 cells were infected with 1000 TCID of the HIV laboratory isolates NL4-3. Infection was inhibited by adding mHSA (final concentration 50 μg/ml). Cell cultures were monitored 5 days after infection by visualization of induced syncytia and cell layer destruction after formalin fixation followed by a standard staining procedure (Hemacolor^®^, Merck). (d-f) To study inhibition of syncytia formation following transfection with pSVATGrev, cells were transfected with the expression plasmid and mHSA was added 30 min later. Syncytia were monitored after 28 h by standard phase contrast microscopy.

For evaluation of virus neutralizing activity, the three HOCl-modified serum albumins were first tested in an MTT-assay (Figure 4a). Using up to 200 μg/ml of HOCl-modified protein, which is at least 10-times of the mHSA concentration necessary to neutralize NL4-3 >95%, we observed no anti-cellular activity in comparison to normal serum albumin. As shown in Figure 4b, virus infection was inhibited by mHSA, and mBSA in a dose-dependent manner to >95% and >90% at a concentration of each 20 μg/ml. Virus inhibition by mMSA was slightly lower and inhibition >90% was observed using 50 μg/ml of mMSA. In addition to viral infection we also tested the inhibition of syncytium formation. As shown in Figure 5, syncytium formation induced by virus strain NL4-3 (Figure 5b) was completely inhibited by mHSA at a concentration 50 μg/ml (Figure 5c). As demonstrated before (Figure 4a) mHSA did not inhibit cell proliferation and, in agreement with this observation, cell morphology and staining of the nucleus proved vitality of the GHOST cells grown in the presence of 50 μg mHSA/ml (Figure 5a, c).

Since syncytium formation is based on the presence of viral envelope and viral receptors on the cellular surface, syncytia can be simply induced by gp160 expression. Therefore, HeLa P4 cells were transfected with gp160 expression vector pSVATGrev. Transfected HeLa P4 cells expose the gp120/gp41 complex on their outer membrane surface and syncytia could be detected after 28 h (Figure 5e). In contrast to non-transfected cells, incubation with mHSA did not show any syncytia (Figure 5d). As demonstrated in Figure 5f, incubation of pSVATGrev transfected cells with mHSA (50 μg/ml) again totally inhibited syncytium formation.

**Figure 6:**
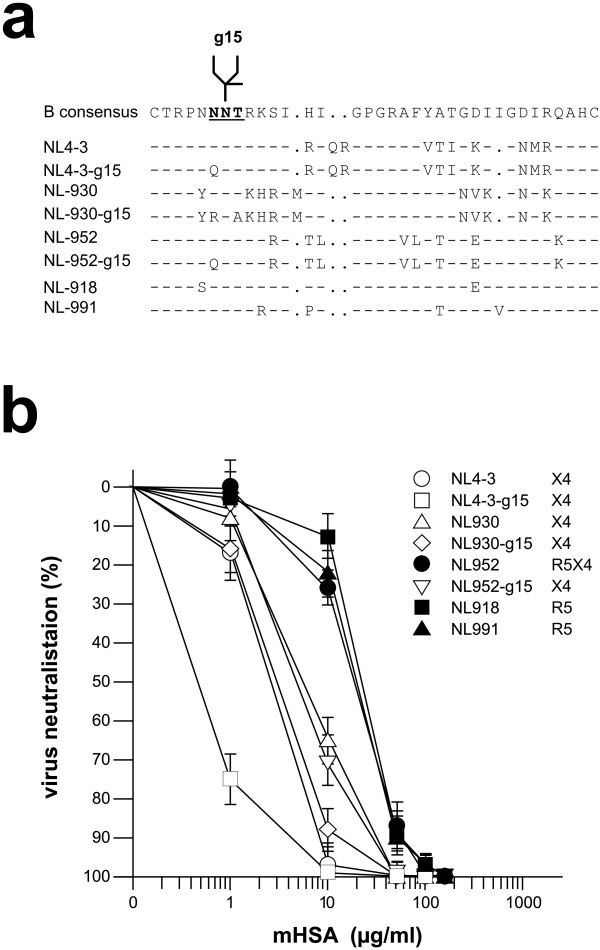
**Inhibition of X4, X4R5 and R5 tropic NL4-3 isolates by mHSA**. (a) V3 loop amino acid sequences of NL4-3 mutants. NNT, recognition site for N-glycosylation. (b) TZM-bl cells were infected with virus supernatant representing an infectious dose of 500 foci/96-well. Infection was inhibited by adding mHSA (molar HSA:HOCl ratio 1:1000) to a final concentration of 1, 10, 50, 100 and 150 μg per ml of culture medium. Viral entry was monitored 2 days after infection by X-gal staining and counting of blue foci. Shown are the averages of triplicate wells. White symbols: X4 tropic viruses. Black symbols X4R5 (NL952) and R5 tropic viruses. The NL4-3 isolates were constructed by an exchange of the NL4-3 V3 loop against V3 loop sequences from primary isolates PI 952, PI-930, PI-918 and PI-991.

Next we investigated the effect of mHSA against infection of V3 loop recombinant NL4-3 viruses of X4, R5X4 and R5 tropism. The tropism was switched due to the exchange of the V3 loop as described earlier [17]. In addition we also tested mHSA against NL4-3 mutants which lack the N glycosylation site g15 within the V3 loop. As shown in Figure 6, all NL4-3 mutants were neutralized efficiently using 100-150 μg mHSA/ml. A significantly higher sensitivity against mHSA was observed for X4 viruses and the viruses lacking N glycan g15.

## Discussion

In the present study, we demonstrated that HOCl-modified serum albumins can bind to LAV gp120, and that HOCl-modified serum albumins possess the ability to efficiently neutralize entry of the NL4-3 laboratory strain. Since the 5'-half of NL4-3 was constructed from NY5 and the 3'-half from LAV the NL4-3 external envelope is identical to the commercial LAV gp120 used in our SPR binding studies.

Binding between the LAV gp120 and our inhibitor was observed in a SPR assay with gp120 covalently linked to a biosensor chip. Binding to gp120 was only monitored with HOCl-modified serum albumin and non-modified serum albumin showed no binding activity. As shown before [16], mHSA binding is also specific to the West Nile virus domain III and does bind to similar domains of Yellow Fever and Dengue-2 virus, respectively. Since mHSA also binds to WNV envelope, we suggest that the binding of HOCl-modified serum albumin to HIV-1 or WNV envelope is neither a highly epitope specific event nor does it mirror a fundamental, yet undiscovered, molecular step of the WNV or HIV-1 entry process. Such non-specific binding events are common in many other pathogen-host interactions. For example malaria parasites use negatively charged chondroitin-sulfate-proteoglycan (CSPG) structures to attach to the membrane surface of their host cells [19]. For HIV-1, heparan-sulfate-proteoglycan (HSPG) is such an attachment factor [20] and heparan sulphates as well as other polyanions are efficient inhibitors of HIV-1 infection [21,22]. Our current hypothesis is that pathogens which coat their core particle with the membrane of the host cell need a simple mechanism to attach to cells possessing an identical membrane structure. This membrane-membrane contact must be mediated by the external envelope glycoprotein complex present in the viral membrane and might be a first non-specific binding event to initiate the next step, binding to specific receptors.

Masking the envelope complex to prevent membrane attachment seems to be a simple mechanism to prevent viral entry. The blockade of CD4 and V3-loop antibody binding by mHSA supports this hypothesis. The masking by mHSA completely inhibited rCD4 binding and partially inhibited the V3-loop antibody binding, indicating that a large and important proportion of the gp120 molecule is masked by mHSA. The surface of gp120 is *per se *covered by complex carbohydrate structures, which represent more than 55% of the gp120 molecular weight. Especially the elimination of the carbohydrate structure g15 within the V3 loop plays an important role in viral entry and these mutants showed higher infectivity. Due to higher infectivity, these viruses can escape from neutralizing antibodies present in human sera [23]. An interesting advantage of mHSA is the ability to neutralize these antibody resistant viruses. A more efficient neutralization of such viruses by mHSA might be the result of the lack of N-glycan g15 that renders the V3 loop more exposed. Such an unmasked V3 loop seems to be a better binding target for HOCl-modified albumins since the binding of V3-loop-specific neutralizing antibodies was blocked by mHSA. Masking the CD4 and V3 loop area by mHSA was also efficient for the blockade of CCR5-specific infection, but 3 times higher levels were necessary compared to the neutralization of CXCR4-viruses. Thus, V3-driven selection for higher infectivity of CXCR4 viruses or the switch from CCR5- to CXCR4-usage is not beneficial for promoting escape from mHSA neutralization. Blocking both coreceptor pathways is an important step to block HIV-1 transmission. In an evolving epidemic, CXCR4-tropism is present in high frequency among all HIV-1 subtypes except subtype C [24,25]. The high frequency of CXCR4-tropism, 86% that was found in subtype A and CRF02_AG in patient samples from West Africa, demonstrates that a topical microbicide has to inhibit CXCR4-viruses frequently present in HIV-1 infected individuals. On the other hand, cervico-vaginal tissue preferentially supports the productive infection by CCR5-tropic viruses [26]. Thus, a microbicide has to block both coreceptor pathways to prevent HIV-1 sexual transmission. The HOCl-modified serum albumins, when applied as a topical microbicide, can be applied in an adequate concentration (>200 μg/ml) that is high enough to block both viruses completely.

In our experiments we added HOCl-modified serum albumin directly into cell culture media. Viral infection assays in cell culture have the advantage that cell culture ingredients such as (i) antioxidants present in fetal calf serum or (ii) free amino acids like methionine or cysteine present in cell culture medium at 0.2 M are present during the test period. In addition, the mHSA samples produced with low amounts of HOCl showed no inhibitory effect indicating that potential traces of taurine or HOCl are not contributing to the inhibitory effect. We also tested our inhibitor in three different neutralization assays using three different cell types (HeLa, GHOST and TZM-bl) which are well established as host cells, widely used in the HIV literature. Since virus is neutralized under all these different cell culture conditions, all the cell culture ingredients will not suppress the antiviral activity of mHSA. Preincubation of cells with mHSA also had no suppressive effect, showing that mHSA is not non-specifically adsorbed by the cells or their different membrane components. Moreover, the normal growth of other viruses like yellow fever and dengue-2 virus in cell cultures, over a period of up to 9 days, containing mHSA [16] indicates that mHSA has no unspecific overall neutralizing activity or toxic activity that inhibits cellular growth and more especially viral production. We have also tested mHSA in other sensitive cell culture systems (Plasmodium falciparum growth in erythrocytes, Lassa-, Marburg-, SARS virus growth) over a time period of 14 days (data not shown). In all these cell culture experiments we observed no inhibitory effect on pathogen growth indicating that mHSA in not a cellular toxin *per se*.

The data implicate that mHSA, or a well defined structure thereof, might be applicable as a drug against viral infection, and that the inoculation of mHSA into infected individuals might overcome adaptive immune tolerance. As was shown in animals, the active immunization of rats with Freund's adjuvant together with a high dose of chlorinated autoantigen induced an immune response against the HOCl-modified protein [27]. Induction of autoantibody might be a risk factor in the direct application of HOCl-modified proteins into body fluids but seems to be no major problem when applied as a topical microbicide. On the other hand, serum albumin is an important carrier for drugs and enzymes. In particular the transport of the enzyme MPO across the endothelial barrier depends on a specific interaction between HSA and MPO [28]. The HSA-binding epitope on MPO was identified as a short linear region (aa 425-454), causing high-affinity binding to HSA. The very close proximity of HSA and MPO implicates that HSA might be highly susceptible for MPO-dependent modifications. This also suggests that MPO-modified serum albumin is a common self antigen and therefore might be tolerated by the immune system.

It is known that HOCl is a powerful oxidant and modifications of protein structure and function are well documented. In published studies, HOCl-induced modifications were analyzed after treatment of peptides or proteins with a 0.5-25-fold molar excess of HOCl [18,29-31]. As shown by Vossmann *et al*. [16] as well as in the present study, an antiviral activity of serum albumins was observed only after treatment of serum albumin with HOCl concentrations above the molar ratio of 1:100. Using lower HOCl concentration, e.g. 1:10 or 1:50 we were unable to detect any antiviral activity in our HIV-1 infection assays. This observation is important and demonstrates that HOCl modifications, which occur under low HOCl conditions [18,29-31] might be totally different to those induced with higher HOCl concentrations as shown here. Another minor aspect is that we have modified albumin at concentrations ≤ 1 mg protein/ml. When serum albumin was treated with HOCl at higher concentrations, as described in the literature (10 mg/ml) [29], we observed no inhibitory activity in our viral infection and syncytium assays (data not shown). Thus, based on our current knowledge, the antiviral activity of serum albumins was induced in diluted aqueous solution and only with a >100-fold molar excess of HOCl.

The modification of proteins by HOCl seems to be a highly complex reaction. Data describing this process are available mainly based on the structure analysis of modified amino acids or peptides at low HOCl concentration. In a report by Salavej *et al*. [31], HSA was treated with HOCl at a 25-fold molar excess, similar to the procedure by Pattison *et al*. [18]. In their analysis, oxygenation was detected for Trp238, Met147, Met353, and Met572, but no chlorination of any of the HSA residues was detected. Using HOBr, Salavej and coworkers detected the incorporation of one or two bromines at Tyr425, but no chlorination of any HSA residues was detected in HOCl treated samples [31]. However, the treatment of amino acid residues with HOCl leads to the documented side chain modifications, but HOCl-modification seems not to be limited to these side chain products. In the context of a linear peptide or protein HOCl-modified side chains undergo intra- or inter-molecular cross linking. Thus, in the context of a complex protein and probably under conditions with excessive concentrations of HOCl, the process is pushed towards the development of complex protein conformations the nature of which is still unknown. One final product of HOCl-treatment seems to be the generation of stable antigenic epitopes on human serum albumin.

Monoclonal antibodies against HOCl-induced epitopes, have been produced. These monoclonal antibodies were mainly raised against oxLDL. One of these monoclonal antibodies was able to bind to the HOCl-induced epitopes on HSA and based on this antibody (clone 2D10G9) HOCl-modified proteins can be detected in human tissue [13-15,29]. It was suggested that the epitope recognized by this monoclonal antibody is of a complex conformational type, but almost nothing is known about the nature of the 2D10G9 epitope. The fact that antibodies can be induced by immunization and HOCl-induced epitopes can easily be detected in human tissue [32] indicates that these HOCl-induced protein structures are chemically and structurally related and seem to be very stable.

Since a 100-fold molar excess of HOCl was necessary to induce the antiviral activity, all the reactions and modifications documented at low HOCl-concentrations might not explain the mechanism by which our HOCl-modified serum albumins inhibit viral entry of HIV-1 as well as West Nile virus. However, our study supports the hypothesis that HOCl might be part of the host defense against pathogens and apart from its direct toxic activity it may have an additional indirect but important activity, the transformation of a protein into an antiviral weapon [16]. From the cell culture experiments of Chase et al. and Klebanoff et al. [11,12], in which activated PMN cells release MPO and HOCl was generated, HOCl was suggested as the HIV-1-killing agent. Based on our results it is conceivable that the generated HOCl reacts with bovine serum albumin, which is usually present in high concentrations in cell culture media, and therefore mBSA would be present during viral infection. As we have now demonstrated, mBSA has a strong antiviral activity and might be also responsible for the antiviral effects observed in PMN cell cultures. The biological relevance of HOCl-modification of serum albumin or other proteins might be questionable since virus replication in vivo is not limited to inflammatory loci, where MPO is expressed in vivo leading to the production of HOCl. To our understanding, the discovery of HOCl-modified serum albumin as an antiviral agent opens a new field for the development of non-toxic agents which can be added to blood products or can be used as a local microbicide to prevent viral transmission. Since the neutralizing activity of HOCl-modified serum albumins was documented against West Nile virus and the HIV-1 NL4-3 mutants, irrespective to their coreceptor type, future work will show how efficient HOCl-modified serum albumins can neutralize other HIV-1 and HIV-2 variants as well as other viral species.

Taken together, the generation of HOCl-modified serum albumins is very simple, cost effective and needs no highly specialized laboratory equipment. We suggest that HIV-1 neutralization by HOCl-modified serum albumins is the result of masking the gp120 molecule. We have shown that the CD4 binding site and other regions, like the V3 loop, are partially masked by HOCl-modified serum albumins. Masking the viral gp120/gp41 envelope complex might be a simple but promising strategy to inactivate HIV-1 and therefore prevent infection when applied, for example, as a topical microbicide.

## Competing interests

The authors declare that they have no competing interests.

## Authors' contributions

SP, MK and BS carried out the virus neutralization experiments. MK developed the procedure for the generation of HOCl-modified proteins and carried out the MTT toxicity assay. MS and MvY produced the HOCl-modified serum albumins. MvY carried out parts of the SPR binding experiments. HS constructed the gp160 expression plasmid and drafted parts of the manuscript. MS carried out the CD4 and antibody binding studies by SPR and ELISA, conceived and coordinated the study and drafted the manuscript. All authors read and approved the final manuscript.
